# Air Pollution, Environmental Violation Risk, and the Cost of Debt: Evidence from China

**DOI:** 10.3390/ijerph19063584

**Published:** 2022-03-17

**Authors:** Aiqun Wang, Ming Zhang, Shuya Zhou

**Affiliations:** School of Business and Management, Jilin University, Changchun 130012, China; aiqun1964@163.com (A.W.); zhou_shuya0304@163.com (S.Z.)

**Keywords:** atmospheric pollution, debt financing cost, environmental penalties, environmental regulatory pressure, heavily polluting firms, state-owned firms, economic contribution

## Abstract

Although a firm’s exposure to air pollution-related risk has become an important factor that creditors cannot ignore in the procedure of lending decision making with the aggravation of air pollution, empirical evidence on whether and how air pollution affects the cost of debt has been relatively scarce. Employing a series of Chinese listed firms from the main board of the Shanghai and Shenzhen Stock Exchanges covering 2014 to 2018, our research responds to this research gap by exploring how air pollution-induced environmental violation risk affects the cost of debt by constructing an assessment system of firms’ environmental violation risk. The results shed light on an issue that firms exposed to higher concentrations of air pollution may suffer a higher environmental violation risk, resulting in a higher debt cost. In addition, a further analysis shows that environmental regulatory pressure and heavily polluting firms enhance the influence of air pollution on the cost of debt, while state-owned firms and firms’ economic contributions weaken the influence of air pollution on the cost of debt. Our research is conducive to highlighting not only the importance of environmental governance for mitigating the cost of debt to the firms exposed to air pollution, but also its importance to creditors exposed to their clients’ environmental violation risk and default risk.

## 1. Introduction

For a long time, some areas in China have sacrificed the environment in exchange for the rapid development of the regional economy [1], causing irreversible damage to the ecological environment. In the last few years, air pollution incidents have occurred frequently in China. In particular, in December 2013, a large-scale air pollution incident occurred in northern China that resulted in large-scale flight delays, the cancellation of outdoor work, and a surge in respiratory diseases [2]. Subsequently, air quality has received increasing public attention as it poses a serious threat to human health, welfare, and psychology [3,4,5]. How to coordinate the relationship between economic growth and environmental protection has become a crucial issue for China’s social development.

With the expansion of the impact of air pollution on the social economy, researchers have gradually become interested in the impact of air pollution on the behavior of capital market participants, which provided us with a crucial and unique idea to explore the relationship between air pollution and the cost of debt. Some frontier literature has noticed that air pollution may increase people’s pessimistic emotions, which leads to a series of aberrant behaviors in capital markets, including risk aversion behaviors and attention-driven buying behaviors among investors [6,7], pessimistic earnings forecasts among securities analysts [8], and more audit efforts for auditors [9]. These unique and innovative studies link air pollution to subsequent abnormal behaviors and outcomes in capital markets, but they have not explored the potential influence of air pollution on the decisions of creditors and the cost of corporate debt. Whether and how air pollution affects the cost of debt is a crucial issue, which is related to the economic benefits and sustainable development of firms. Our study explores the effect of air pollution on the cost of debt in depth based on large-sample data to provide empirical evidence for whether and how air pollution affects creditors’ decisions.

Public concern regarding the ecological environment has increased along with air pollution, stimulating government regulators to formulate stricter environmental regulations [10]. Firms are not only powerful drivers of economic growth, but also major producers of air pollution. The Chinese government has passed a great deal of powerful environmental regulations aimed at reducing the pollutant gas emissions of firms, including environmental protection interview systems and the Blue Sky Defense Campaign. Undoubtedly, these measures severely punish the polluting behaviors of firms, which leads many heavily polluting firms to face a huge environmental violation risk [11]. Firms’ exposure to environmental violation risk may increase the uncertainty risk in its current and future cash flows and, ultimately, increase the likelihood of the default [10,12]. With the increasing pressure of environmental supervision, firms’ exposure to environment-related risk has become an important factor that creditors cannot ignore in the procedure of lending decision making [10,13,14], which means that more and more firms are facing the severe challenge of financing constraints caused by air pollution [15,16]. Therefore, whether air pollution affects the cost of debt through its effect on environmental violation risk is worthy of exploration.

Empirical evidence from China can provide an ideal natural setting for this study. First, China’s vast geographical area includes different climatic conditions, which can be more conducive for us to identify the potential impact of different levels of air pollution on corporate debt cost. Second, Chinese empirical evidence provides us with data on firms under discrepant conditions of environmental regulation, industry characteristics, ownership, and economic contribution that facilitate the analysis of the moderating roles of these heterogeneous factors in the impact of air pollution on the cost of debt.

Employing the listed firms from the main board of the Shanghai stock exchange (SSE) and Shenzhen stock exchange (SZSE) from 2014 to 2018 as the research sample, we thoroughly explore whether and how air pollution surrounding the cities where the firms are located becomes an important factor affecting the cost of debt. The results show that air pollution in the cities of the firms are significantly positively associated with the cost of debt, and environmental violation risk is the mechanism of air pollution affecting the cost of debt. This reveals that firms exposed to higher concentrations of air pollution may suffer a higher risk of environmental violations, which promotes the creditors’ pessimistic assessment of firms’ default prospects and results in a higher cost of debt. Our main regression result remains robust after a range of sensitivity tests and endogenous tests. A further analysis shows that the environmental regulatory pressure and heavily polluted firms enhance the influence of air pollution on the cost of debt, while state-owned firms and firms’ economic contributions weaken the influence of air pollution on the cost of debt.

The research contributions we consider may include the following parts: First, we fill in the gaps in the existing literature on the relation between air pollution and the cost of debt. This research focuses on the effect of air pollution on the cost of debt, which not only extends the related literature on the economic outcomes of air pollution, but also enriches the research paradigm of “environmental conditions–decision making”. Second, our research provides deeper insights into how air pollution affects creditors’ decisions and corporate debt cost from the perspective of environmental violation risk. Although some scholars have attempted to analyze the restrictive effect of air pollution on corporate debt financing from the mechanism of credit risk and financial uncertainty [15,16], they ignored the mechanism of environmental violation risk, which is the fundamental mechanism by which air pollution affects the cost of debt. Additionally, few studies have comprehensively measured the environmental violation risk of listed firms. Our research responds to these research gaps by clarifying the internal mechanism of air pollution affecting the cost of debt by constructing an evaluation system of firms’ environmental violation risk based on the scoring standard for firms’ environmental violations formulated by the Chinese local government. Third, our study provides firms with useful insights for forecasting creditors’ responses and decision making in the face of air pollution based on diverse conditions that include regional environmental supervision, industry characteristics, ownership, and firms’ economic contribution. Specifically, our research reminds the firms subject to strict environmental supervision, heavily polluting firms, non-state-owned firms, and firms with low economic contributions to be more vigilant against the environmental risk hidden in air pollution, which complements the existing literature on the environmental violation risk of firms to a certain extent [17,18,19].

## 2. Literature Review and Hypothesis Development

With the frequent occurrence of global haze events, the severity of air pollution has gradually attracted extensive attention. As a typical indicator of environmental conditions, air pollution observably affects the judgment and decision making of capital market participants, which is gradually being recognized by scholars. Levy and Yagil [6] and Lepori [20] propose that air pollution may deteriorate investors’ emotions and intensify their risk aversion, which leads to a negative impact on the subsequent stock returns. Coincidentally, Huang et al. [7] confirmed that air pollution makes investors more prone to the disposition effect, attention-driven buying behavior, and excessive trading, which may contribute to poor trading performance. Wu et al. [1] also verified that the fluctuation of investor sentiment related to air pollution is an important reason for the abnormal stock price. Subsequently, some scholars even further extended the relationship between air pollution and the decision making of capital market participants to analysts’ earnings forecast and auditors’ pessimistic bias [8,9]. However, few studies discuss whether and how atmospheric pollution influences the judgment and decision making of the lenders compared to stock investors, which is closely related to the debt financing and sustainable development of firms.

In the following analysis, we deeply explored (1) the impact of air pollution on the cost of debt and the mediating effect of environmental violation risk on this impact and (2) the heterogeneous factors affecting the relationship between air pollution and debt cost.

### 2.1. Influence of Air Pollution on Cost of Debt and Mechanism of Environmental Violation Risk

Recently, the frequent occurrence of serious air pollution, such as high concentrations of haze, has aroused widespread concern and pessimistic expectations among the public regarding a plummeting air quality and environmental livability, since air pollution has gradually become the main culprit of many diseases [21,22,23]. Meanwhile, these public concerns and pessimistic expectations concerning air pollution are also potential forces to promote government regulators to implement sterner environmental regulatory measures to severely punish environmental violations [24,25]. Firms are not only the main driving force of economic development, but also resource consumers and manufacturers of environmental pollution. In particular, heavily polluting firms may face a greater environmental violation risk due to the penalty of pollution discharge by environmental supervisions in areas with serious air pollution [11], which is a crucial risk signal to creditors and other stakeholders in circumstances of asymmetric environmental information. The financial losses of environmental violation risk for firms are quite serious, which may increase the uncertainty of their future cash flows [10,12,26,27], threaten viability [28], and even increase the default risk or bankruptcy risk of firms [12,18]. Generally, firms with higher exposure to environmental violation risk face higher credit risk [14]. In response, creditors, including banks and other lending institutions, cannot ignore the environmental risk related to air pollution in the process of lending decision making [13,28,29] and prevent the air pollution-related default loss by revising debt contracts, such as raising the bond yields and interest rate as a response [10,12,30]. Tan et al. [16] and Tan et al. [15] also confirmed that firms in areas with serious air pollution face more significant debt financing constraints in the process of negotiation with banks due to a higher credit risk and financial uncertainty related to the environment. Consequently, we expect that firms located in areas with serious air pollution face a higher risk of environmental violations, and, thus, they need to afford higher debt costs in the process of debt financing. The above theoretical analysis led to the following hypothesis:

**Hypothesis** **1a** **(H1a).** *Higher concentrations of air pollution cause firms to bear a higher cost of debt.*

**Hypothesis** **1b** **(H1b).** *Environmental violation risk is the mechanism for the effect of air pollution on the cost of debt, that is, air pollution increases the cost of debt through its effect on environmental violation risk.*

### 2.2. Moderating Role of Environmental Regulatory Pressure

The original pressure for firms to carry out environmental management comes from strict governmental regulations, and its purpose is to maintain legitimacy [31,32,33]. Strict environmental policies threaten the legitimacy of polluting firms and bring administrative penalties or legal proceedings against polluting firms [34,35], which bring losses to the reputation and market valuation of firms [36]. During the 11th five-year plan period, Chinese local governments continued to strengthen the implementation of environmental policies, which was represented by the inclusion of regional air pollution control in the assessment system of local governments [17,37]. Since 2014, the Ministry of Environmental Protection of China has implemented a new policy of interviewing local government managers. In 2018, the State Council issued the three-year action plan for winning the Blue Sky Defense Campaign, focusing on strengthening the supervision of regional air quality. As a result of a stricter environmental regulation enforcement, the environmental violations of firms due to poor environmental performance have gradually attracted serious attention from investors and have begun to affect investors’ assessments of corporate risk [38]. We speculate that the environmental violation risk related to air pollution is higher for firms located in these regions with strict environmental supervision. Creditors are more sensitive to environmental regulatory compliance, environmental violation risk, and the legitimacy status of firms subject to strict environmental supervision. In particular, OECD countries have had a strong risk awareness of climate change for a long time, which has prompted them to adopt strict environmental regulations in response to the threat of climate change [39]. Firms in OECD countries may face a higher environmental violation risk related to air pollution, which results in higher costs of debt.

**Hypothesis** **2** **(H2).** *The effect of air pollution on the cost of debt is strengthened by environmental regulatory pressure.*

### 2.3. Moderating Role of Firms’ Industry Characteristics

Prior studies have demonstrated that industry characteristics are a critical factor affecting the investors’ attention to firms’ environmental issues. Firms in environmentally sensitive industries have a higher tendency to engage in polluting activities and noncompliance with environmental regulations, and investors are more likely to perceive the environmental risk characteristics of these firms [38]. In particular, the announcement of new environmental regulations reduces investors’ aspiration to invest in heavily polluting stocks, which eventually leads to a relatively poor stock return performance of heavily polluting firms in a short period [11]. Konar and Cohen [40] demonstrated that, driven by environmental responsibility, some investors have consciously penalized heavily polluting firms by raising the cost of capital. Thus, we expect that when cities are covered in heavy air pollution, creditors would pay more attention to the environmental problems of heavily polluting firms in these areas and worry about the risk of environmental violations of these heavily polluting firms.

**Hypothesis** **3** **(H3).** *The effect of air pollution on the cost of debt is strengthened by heavily polluting firms.*

### 2.4. Moderating Role of Firms’ Ownership

Under the special circumstances of China, the ownership of firms is often regarded as an important factor affecting environmental governance issues. In China, state-owned firms often belong to important industries related to the national economy and the people’s livelihood and bear the important functions of maintaining national security, economic stability, industrial leadership, and public services. In order to ensure the state-owned economy’s control over these key industries, the government must hold a certain proportion of shares in these key firms and appoint specific officials as senior managers of state-owned firms [41]. Thus, ownership is regarded as a strong political link between firms and government [42]. Political connection not only brings more advantages to state-owned firms, such as more investment funds, tax incentives, lower financing constraints, and capital cost [43,44,45,46], but also brings some obstacles to the implementation of environmental supervisions by local governments [17,38]. When local governments implement environmental supervision, the political connection can always protect state-owned firms from punishments due to environmental violations to a great extent. Therefore, the regional political protection of heavy polluters has often been accused of being a barrier for the enforcement of environmental regulations [47]. Zhou et al. [18] also demonstrated that creditors more carefully assess the carbon risk of private firms and adopt stricter approval standards in the process of reviewing loans. We expect that when air pollution occurs, political connection can play a role as an umbrella to minimize the penalties for environmental violations doled out to state-owned firms, reducing the sensitivity of creditors to the environmental risk of state-owned firms.

**Hypothesis** **4** **(H4).** *The effect of air pollution on the cost of debt is weakened by state-owned firms.*

### 2.5. Moderating Role of Firms’ Economic Contribution

For a long time, it has generally been acknowledged that the importance of economic development is higher than environmental protection in Chinese society, which has led to weak economic punishments for environmental violations among Chinese firms [48]. Under the current administrative system in China, environmental policies are mainly formulated by the central government, while the implementation functions of environmental policies are mostly carried out by local governments, which are the main body of environmental governance and supervision. However, in the process of implementing environmental policies, local governments often fail to supervise the environmental violations of local firms. As Wang and Wheeler [49] noted, the bargaining power of firms is an important factor affecting the effectiveness of governments’ environmental supervision. The tax contributions from firms’ business activities are conducive to promoting local economic growth and realizing the promotion goals of local officials. If a firm is a major contributor to local economic development, it may have stronger bargaining power when it comes to environmental supervision. Therefore, under the condition of serious information asymmetry in environmental problems, for the consideration of economic development and political performance objectives, local managers are likely to tolerate the environmental violations of heavily polluting firms that provide significant contributions to local economies and taxation at the expense of environmental protection [17]. We expect that an economically important firm has stronger negotiating abilities when it comes to environmental supervision, effectively reducing the administrative penalty they may suffer in air pollution events and reducing the sensitivity of creditors to the environmental violation risk.

**Hypothesis** **5** **(H5).** *The effect of air pollution on the cost of debt is weakened by firms’ economic contribution.*

## 3. Research Design

### 3.1. Sample Selection

We selected a sample of Chinese listed firms from the main board of the SSE and SZSE from 2014 to 2018. The SSE and SZSE are the distinguished stock exchanges in China. Listed firms from the main board of the SSE and SZSE are generally characterized by a high market share, large scale, and strong comprehensive strength, and their environmental performance and environmental violations are more susceptible to the attention of government regulators, creditors, and other stakeholders. Consequently, these firms served as the focus for our research to analyze the possible relationship between air pollution and the cost of debt. The reason for selecting this time period was that China’s air quality evaluation system has changed to a great extent since 2014. Therefore, this paper selected the air quality data of 2014 and later to ensure the consistency of the calculation caliber of air pollution degree. Air pollution data of cities in China and financial data of the listed firms were collected from the China Stock Market and Accounting Research (CSMAR) database. We obtained related macroeconomic data, including the regional GDP growth rate and proportion of secondary industry, from the China Statistical Yearbooks. Furthermore, regional climate data, including wind speed and annual rainfall, were manually collected from the meteorological database on the website of greenhouse data sharing platform and the Statistical Yearbooks of China’s provinces. Data related to firms’ environmental violations were manually collected from the firms’ environmental supervision records on the website of the Institute of Public and Environmental Affairs (IPE). Next, we eliminated the sample firms that met the following conditions according to the conventional practice to ensure the reliability of the research results: (i) financial firms (since the accounting standards of the financial industry are quite different from those of other industries, we excluded financial listed firms from the full sample); (ii) firms with missing key financial data; (iii) firms with serious financial abnormalities or delisting risk (ST or *ST). We ultimately retained 3993 firm-year observations after a prudent screening process. Finally, all continuous variables were winsorized at the 1% level to eliminate the effects of extreme values.

### 3.2. Main Variable Descriptions

#### 3.2.1. Dependent Variable: Cost of Debt (Cost)

In terms of the measurement of the core dependent variable, we used the firm’s interest expenditure rate as the proxy of the cost of debt similar to prior studies on the listed firms, which was measured as the total interest expense divided by the average interest-bearing debt [18,50,51,52].

#### 3.2.2. Independent Variable: Air Pollution (Air)

Before 2013, the Ministry of Environmental Protection of China (MEPC) issued the air pollution index (API), which is an index for calculating and comprehensively evaluating air quality conditions according to the concentration of five main pollutants, such as PM_10_ [1]. Subsequently, after 2013, a severe haze occurred in many cities in China, but the API did not contain the index of PM_2.5_, which is the main pollutant component of haze. Therefore, the MEPC used the air quality index (AQI) to replace the original air pollution index (API). According to the technical regulations of ambient AQI issued by the MEPC, the AQI is evaluated based on six atmospheric pollutants, including SO_2_, NO_2_, PM_10_, PM_2.5_, CO, and O_3_, which can more comprehensively evaluate the air quality of cities in China. Therefore, similar to prior studies on the Chinese atmospheric pollution [1,8,53], we used the mean value of the daily AQI of the cities where the firms were located in the current year to comprehensively measure the degree of air pollution. Larger values of average daily AQI indicated that firms were exposed to more severe air pollution.

#### 3.2.3. Mediating Variable: Environmental Violation Risk (Violation)

The variable violation refers to the environmental violation risk of firms. Although Dobler et al. [54] and Zhou et al. [18] used ordered variables to measure the environmental violation risk of firms, we still insist that this measurement is too simple to accurately and comprehensively evaluate firms’ environmental violation risk, which may lead to biased research conclusions. Some local governments in China have issued the measures for the environmental credit evaluation of firms, which recorded detailed scoring standards for environmental violations of firms, including information on environmental violations and their corresponding scoring. Therefore, combined with the scoring standard of firms’ environmental violations attached to the firms’ environmental credit evaluation criteria issued by China’s local governments, we constructed an assessment system of corporate environmental violation risk as represented in detail in Table 1. The specific process of calculating the environmental violation risk of firms in this research was as follows: First, we summed up the punishment scores corresponding to each environmental violation of a firm in the current year to obtain the severity of environmental violations (Severity). In order to more accurately identify the impact of local air pollution concentration on the environmental violation risk of firms, we excluded the records of environmental violations of affiliated firms whose regions were inconsistent with where the listed firms’ offices were located. Secondly, Ln (Severity + 1) was calculated to measure the firms’ environmental violation risk (Violation).

#### 3.2.4. Control Variables

Referring to Zhou et al. [18] and Shailer and Wang [51], we considered a series of control variables that were used in the literature and were related to the cost of debt. First, we controlled for size (Size), which was calculated as the natural logarithm of the firm’s total assets. Larger firms may have more mortgage assets, which means they have a stronger risk resistance and lower debt cost. The second control variable was ownership of firms (State). State-owned firms usually have debt guaranteed and financial support provided by the government, and their default risk and debt cost are correspondingly lower. The third control variable was leverage (Lev), which was calculated as the ratio of total debt to total assets. A higher asset liability ratio means a higher possibility of corporate debt default, and creditors need more risk premiums as compensation. The fourth control variable was the interest coverage ratio (Ic), which was calculated as the ratio of earnings before interest and tax (EBIT) to interest expense. The interest coverage ratio represents the firm’s ability to pay interest, and the interest coverage ratio is negatively associated with the cost of debt. The fifth control variable was the fixed assets ratio (Fix), which was calculated as the ratio of total fixed assets to total assets. Firms with more fixed assets usually have a lower asset liquidity, lower capital turnover rates, and weaker operating capacities, which result in a higher cost of debt financing. The sixth control variable is the rate of return on total assets (Roa), which represents the profitability of firms. Firms with a higher Roa have a stronger profitability and solvency. Thus, Roa is negatively associated with corporate debt cost. The seventh control variable was growth opportunity (Growth), which was measured as the revenue growth rate. Firms with higher growth opportunities are generally expected to have a higher default risk. The eighth control variable was the operating cash flow of firms (Cfo), which was calculated as the ratio of operating cash flow to total assets. Firms with an adequate cash flow from operations usually have a lower debt cost. We also controlled some regional macroeconomic variables expected to impact the cost of debt: the provincial GDP growth rate (Gdp) and proportion of provincial secondary industry (Second). Specifically, the pursuit of GDP growth is an important reason for the government to reduce environmental supervisions for firms. At the same time, the proportion of secondary industry is a reflection of industrialization, which is typically accompanied by the environmental deregulation of local governments [55]. From this, GDP growth and the proportion of secondary industry may be associated with the environmental violation risk of firms and could ultimately affect the cost of debt. Furthermore, we also considered the possible impact of annual, industrial, and provincial fixed effects on the empirical results. Detailed variable definitions are listed in Table 2.

### 3.3. Research Model

In order to verify the main theoretical hypothesis that air pollution surrounding cities where the firms were located increased their cost of debt, we used the OLS method to construct the multiple linear regression model seen in Equation (1). The principal coefficient of interest was $\alpha_{1}$, which was expected to be positive. In the robust test, endogeneity-corrected regression methods (two-stage least squares regressions and the propensity score matching method) were used.

(1) $$\begin{array}{l} Cost_{i,t}=\alpha_{0}+\alpha_{1}Air_{i,t}+\alpha_{2}Size_{i,t}+\alpha_{3}State_{i,t}+\alpha_{4}Lev_{i,t}+\alpha_{5}Ic_{i,t}+\alpha_{6}Fix_{i,t}+\alpha_{7}Roa_{i,t}\\ +\alpha_{8}Growth_{i,t}+\alpha_{9}Cfo_{i,t}+\alpha_{10}Gdp_{i,t}+\alpha_{11}Second_{i,t}+\sum Year+\sum Industry+\sum Province+\varepsilon \end{array}$$

According to the previous theoretical analysis, we conjectured that air pollution increases the negative expectations of the public for atmospheric quality, which then encourages the government regulatory authorities to formulate stricter environmental regulatory policies and punish the environmental violations of firms. Firms exposed to heavy air pollution are confronted with more severe environmental violation risks, which increases the debt cost of firms. Referring to the mediating effect test of Baron and Kenny [56], we constructed Equations (2) and (3) by the OLS method to test whether air pollution increased the cost of debt through its effect on environmental violation risk.

(2) $$\begin{array}{l} Violation_{i,t}=\alpha_{0}+\alpha_{1}Air_{i,t}+\alpha_{2}Size_{i,t}+\alpha_{3}State_{i,t}+\alpha_{4}Lev_{i,t}+\alpha_{5}Ic_{i,t}+\alpha_{6}Fix_{i,t}+\alpha_{7}Roa_{i,t}\\ +\alpha_{8}Growth_{i,t}+\alpha_{9}Cfo_{i,t}+\alpha_{10}Gdp_{i,t}+\alpha_{11}Second_{i,t}+\sum Year+\sum Industry+\sum Province+\varepsilon \end{array}$$

(3) $$\begin{array}{l} Cost_{i,t}=\alpha_{0}+\alpha_{1}Air_{i,t}+\alpha_{2}Violation_{i,t}+\alpha_{3}Size_{i,t}+\alpha_{4}State_{i,t}+\alpha_{5}Lev_{i,t}+\alpha_{6}Ic_{i,t}\\ +\alpha_{7}Fix_{i,t}+\alpha_{8}Roa_{i,t}+\alpha_{9}Growth_{i,t}+\alpha_{10}Cfo_{i,t}+\alpha_{11}Gdp_{i,t}+\alpha_{12}Second_{i,t}+\sum Year\\ +\sum Industry+\sum Province+\varepsilon \end{array}$$

In order to further test the moderating role of environmental regulatory pressure on the relationship between air pollution and the cost of debt, we introduced the intersection term of environmental regulatory pressure and air pollution as shown in Equation (4). Since 2008, the IPE and NRDC (Natural Resources Defense Council) have continuously published the Pollution Information Transparency Index in China (PITI), which is an index system for evaluating the information disclosure of pollution source supervision in key environmental protection cities in China. Specifically, the PITI takes more than 100 critical cities in China as the evaluation object every year and comprehensively scores and evaluates the environmental supervision pressure of these cities according to eight items, such as the publicity of the daily exceeding standard of pollution sources and illegal record information, the publicity of centralized remediation information of pollution sources, and the publicity of cleaner production audit information. As such, we used the PITI to measure the environmental regulatory pressure suffered by the sample firms. The coefficient $\alpha_{3}$ in Equation (4) was expected to be significantly positive if Hypothesis 2 was confirmed.

(4) $$\begin{array}{l} Cost_{i,t}=\alpha_{0}+\alpha_{1}Air_{i,t}+\alpha_{2}PITI_{i,t}+\alpha_{3}AQI\times PITI_{i,t}+\alpha_{4}Size_{i,t}+\alpha_{5}State_{i,t}+\alpha_{6}Lev_{i,t}+\alpha_{7}Ic_{i,t}\\ +\alpha_{8}Fix_{i,t}+\alpha_{9}Roa_{i,t}+\alpha_{10}Growth_{i,t}+\alpha_{11}Cfo_{i,t}+\alpha_{12}Gdp_{i,t}+\alpha_{13}Second_{i,t}+\sum Year\\ +\sum Industry+\sum Province+\varepsilon \end{array}$$

In order to further test the moderating role of firms’ industry characteristics on the relationship between air pollution and the cost of debt, we introduced the intersection term of firms’ industry characteristics and air pollution as shown in Equation (5). Specifically, we extracted the list of heavily polluting firms according to the classified management directory of environmental protection verification industry of listed firms issued by the MEPC. Next, we created a classified variable, Polluted, which represented the firms’ industry characteristics and assigned it to 1 if the firm belonged to a heavily polluting industry, while the others were 0. The coefficient $\alpha_{3}$ in Equation (5) was expected to be significantly positive if Hypothesis 3 was confirmed.

(5) $$\begin{array}{l} Cost_{i,t}=\alpha_{0}+\alpha_{1}Air_{i,t}+\alpha_{2}Polluted_{i,t}+\alpha_{3}Air\times Polluted_{i,t}+\alpha_{4}Size_{i,t}+\alpha_{5}State_{i,t}+\alpha_{6}Lev_{i,t}\\ +\alpha_{7}Ic_{i,t}+\alpha_{8}Fix_{i,t}+\alpha_{9}Roa_{i,t}+\alpha_{10}Growth_{i,t}+\alpha_{11}Cfo_{i,t}+\alpha_{12}Gdp_{i,t}+\alpha_{13}Second_{i,t}+\sum Year\\ +\sum Industry+\sum Province+\varepsilon \end{array}$$

In order to further test the moderating role of firms’ ownership on the relationship between air pollution and the cost of debt, we introduced the intersection term of firms’ ownership and air pollution as shown in Equation (6). As mentioned above, we assigned the variable State to 0 if the affiliation of the actual controller of a firm was the state, and 1 otherwise. The coefficient $\alpha_{3}$ in Equation (6) was expected to be significantly positive if Hypothesis 4 was confirmed.

(6) $$\begin{array}{l} Cost_{i,t}=\alpha_{0}+\alpha_{1}Air_{i,t}+\alpha_{2}State_{i,t}+\alpha_{3}Air\times State_{i,t}+\alpha_{4}Size_{i,t}+\alpha_{5}Lev_{i,t}+\alpha_{6}Ic_{i,t}\\ +\alpha_{7}Fix_{i,t}+\alpha_{8}Roa_{i,t}+\alpha_{9}Growth_{i,t}+\alpha_{10}Cfo_{i,t}+\alpha_{11}Gdp_{i,t}+\alpha_{12}Second_{i,t}+\sum Year\\ +\sum Industry+\sum Province+\varepsilon \end{array}$$

In order to further test the moderating role of firms’ economic contribution on the relationship between air pollution and the cost of debt, we introduced the intersection term of firms’ economic contribution and air pollution as shown in Equation (7). Following Liu et al. [17], we measured the economic contribution of a firm by its tax expenditure as a percentage of the total tax revenue in that province. The variable Tax refers to a firm’s economic contribution. The coefficient $\alpha_{3}$ in Equation (7) was expected to be significantly negative if Hypothesis 5 was confirmed.

(7) $$\begin{array}{l} Cost_{i,t}=\alpha_{0}+\alpha_{1}Air_{i,t}+\alpha_{2}Tax_{i,t}+\alpha_{3}Air\times Tax_{i,t}+\alpha_{4}Size_{i,t}+\alpha_{5}State_{i,t}+\alpha_{6}Lev_{i,t}\\ +\alpha_{7}Ic_{i,t}+\alpha_{8}Fix_{i,t}+\alpha_{9}Roa_{i,t}+\alpha_{10}Growth_{i,t}+\alpha_{11}Cfo_{i,t}+\alpha_{12}Gdp_{i,t}+\alpha_{13}Second_{i,t}\\ +\sum Year+\sum Industry+\sum Province+\varepsilon \end{array}$$

## 4. Empirical Results

### 4.1. Descriptive Statistics

Table 3 presents a sample distribution by the provinces where the firms were located and mean values of the AQI during our sample period. In our research sample, the province with the largest number of sample firms was Beijing, followed by Zhejiang and Guangdong. The province with the worst air quality was Hebei, with a mean AQI of 124.1973 during the sample period, and the province with the best air quality was Hainan, with a mean AQI of 46.4630 during the sample period. Combined with the technical regulation on ambient AQI issued by the MEPC in 2012, an AQI exceeding 100 was considered polluted, indicating that some central and eastern provinces in China had an unhealthy air quality.

Table 4 shows the descriptive statistical results of the main research variables. The cost of debt financing varied from 0.0004 to 0.0773, which showed that there were prominent differences in debt financing cost between different firms from the sample. Moreover, the mean and median values of debt financing cost from our sample were 0.0320 and 0.0314, respectively, which were much lower than 0.065 and 0.061 [18], indicating that the debt cost of high-carbon firms was higher to a certain extent. The mean value of our sample cities’ daily AQI was 88.7361, indicating that the air quality of the cities where most of the sample firms were located was good according to the MEPC. However, the maximum value of AQI was 142, indicating that some firms were still exposed to a polluted atmosphere. In addition, the mean value of the leverage (Lev) was 0.5173, confirming that most of the firms from the sample preferred debt financing.

The results in Table 5 show that the highest correlation coefficient was 0.4049 between Roa and Cfo. In addition, given that the coefficients were all less than 0.8, it could be considered that there was no serious multicollinearity among regression model variables.

### 4.2. Regression Results of the Influence of Air Pollution on the Cost of Debt

Columns (1) and (2) of Table 6 present the regression results of the influence of air pollution on the cost of debt. Column (1) shows the regression results without control variables of firms’ characteristics and regional economic characteristics. As predicted in the prior hypothesis, the coefficient of air pollution (Air) in column (1) was positive and statistically significant at the 5% level, supporting our hypothesis that firms exposed to more serious air pollution in their cities have to bear higher debt costs. Column (2) added control variables representing firms’ characteristics and regional economic characteristics. The coefficient of Air in column (2) still remained statistically significant at the 1% level, demonstrating that air pollution was significantly positively associated with the cost of debt (Cost) after firms’ characteristics and regional economic characteristics were considered. The positive effect of air pollution surrounding the cities where the firms were located on the cost of debt demonstrated that firms exposed to more serious air pollution were more likely to face a higher risk of environmental violations, which correspondingly increased the default risk of firms. Under such circumstances, creditors can only raise the debt cost of firms to reduce air pollution-related default losses. Thus, the empirical results supported Hypothesis 1a. It is worth noting that the cost of debt includes multiple influencing factors that cannot be captured completely. Based on the existing literature, the control variables in our research models largely included the factors that may affect the cost of debt. The adjusted-R^2^ value in model (2) equaled 0.3318, which was similar to the 0.334 of Tan et al. [16] and the 0.373 of Zhou et al. [18], and was slightly higher than the 0.283 of Jung et al. [10] and the 0.105 of Chen et al. [9]. It indicated that the fitting degree of the model was acceptable. In addition, we also employed an analysis of variance to evaluate the overall goodness-of-fit of models (1) and (2). The results of the analysis of variance in Table 7 revealed that the F-statistics of models (1) and (2) were all significant at the 1% level, which indicated that the regression models had statistical significance as a whole.

Consistent with Shailer and Wang [51], the coefficient of firms’ ownership (State) was statistically significant, confirming that the ownership of firms is a critical factor affecting the cost of debt in the Chinese context. In other words, the cost of debt financing of firms under government control was significantly lower than that of firms under private control. The coefficients for Lev, Ic, Fix, and Growth were all significant at the 1% level, confirming that firms with higher leverage, a lower interest coverage, higher fixed assets ratio, and higher growth opportunities were more likely to be able to afford higher costs of debt.

### 4.3. Mechanism of Environmental Violation Risk

Columns (3) and (4) of Table 6 reflect the regression results on the mechanism of environmental violation risk. The coefficient of Air in column (3) of Table 6 was positive and statistically significant at the 1% level, confirming that air pollution surrounding the cities where the firms were located significantly increased the firm’s environmental violation risk (Violation). The coefficient of Violation in column (4) of Table 6 was also significantly positive, which meant that air pollution increased the firm’s cost of debt through its effect on the environmental violation risk. Therefore, the mediating effect test confirmed Hypothesis 1b (environmental violation risk was the mechanism of air pollution affecting the cost of debt). In addition, the coefficient of Air in column (4) remained significant, indicating that the environmental violation risk was the partial mediating variable between air pollution and the cost of debt. To guarantee the reliability of the conclusion, our research also carried out a Sobel test. The Z statistic was 1.7600 and was significant at the level of 10%, which proved the inference of the mediating effect of environmental violation risk again.

### 4.4. Robustness Test

#### 4.4.1. Sensitivity Test for Measurement of Air Pollution and the Cost of Debt

In the previous regression analysis, we used the AQI to measure the air pollution concentrations of the cities where the firms were located; here, we needed to use other indicators to replace the AQI so as to ensure the robustness of the regression results. Firstly, considering that PM_2.5_ was the most important component of atmospheric pollutants, we used the method developed by Chen et al. [9] to measure the air pollution by average PM_2.5_ concentration (PM_2.5) in cities where the firms were located. Next, MEPC divided the AQI once into six levels according to the severity of pollution: excellent (0 < AQI ≤ 50), good (50 < AQI ≤ 100), slightly polluted (100 < AQI ≤ 150), moderately polluted (150 < AQI ≤ 200), heavily polluted (200 < AQI ≤ 300), and severely polluted (AQI > 300). Therefore, referring to Dong et al. [8], we changed the AQI into a classified variable (AQI_grade) according to the classification standard of MEPC for the air quality grade. We employed PM_2.5 and AQI_grade to remeasure air pollution. The results of the sensitivity test of air pollution are presented in columns (1) and (2) of Table 8. The coefficients of PM_2.5 and AQI_grade were 0.0001 and 0.0016, which were significantly positively associated with the Cost, and they were coincident with the results shown in Table 6. This showed that our inference remained robust after replacing the measurement of air pollution. 

Following previous studies on the Chinese firms [57,58], we applied the total interest expense divided by the average total debt (Expense) as an alternative measurement for the cost of debt and re-estimated Equation (1). The results of the sensitivity test of the cost of debt are presented in columns (3), (4), and (5) of Table 8. The coefficients of Air, PM_2.5, and AQI_grade were all significantly positively associated with the Expense, and they were consistent with those shown in Table 6.

#### 4.4.2. Two-Stage Least Squares (2SLS) Analysis

From a more rigorous perspective, this study may have had some endogenous problems that needed to be solved. The first problem was that there may have been a two-way causal relationship between air pollution and the cost of debt. Previous studies have shown that debt financing can have an impact on corporate performance or corporate value [59,60,61], and a higher cost of debt capital inevitably increases the operating pressure and earnings pressure of firms. Firms with earnings pressure are more motivated to emit polluting gases [17], which, inevitably, aggravates air pollution. In addition, there may be the problem of missing variables in the research, since there were other potential factors affecting the cost of debt, including the regional legal environment [62,63,64], monetary policy [65], internal control quality [66], and other factors. Consequently, we employed a 2SLS regression with instrumental variables to mitigate potential endogenous problems.

Past studies have found that air pollution is closely related to meteorological factors, including rainfall, wind speed, wind direction, air temperature, etc. [67,68]. At the same time, these meteorological factors were unlikely to have an influence on the cost of debt. Therefore, rainfall (Rain) and wind speed (Wind) in the cities where the firms were located were selected as instrumental variables of air pollution in this paper. As reflected in column (1) of Table 9, Rain and Wind were both significantly related to Air. Under the 2SLS method, the coefficient of Air in column (2) was still significantly positive, which reflected that the regression results of 2SLS also supported the main assumptions of this paper. Furthermore, in order to examine the effectiveness of instrumental variables, this paper implemented a weak instrumental variable test and over-identification test. The LM statistic was significant at the 1% level, demonstrating that the problem of the underestimation of instrumental variables could be eliminated. The *p*-value of the Sargan statistic was 0.2462, which indicated that there was no overestimation problem either. In addition, the Shea’s partial R^2^ was 0.0290, and its F statistic and the *p*-value of its F statistic were 36.6104 (more than 10) and 0.0000, respectively. We could, thus, insist that there was no weak instrumental variable. Overall, this result re-confirmed that air pollution enhanced the cost of corporate debt.

#### 4.4.3. Propensity Score Matching Estimation (PSM)

Finally, for the purpose of alleviating the endogenous problems caused by selection bias, we also used the propensity score matching (PSM) method to construct new samples to re-test our major assumptions. Specifically, we first selected the experimental group and the control group. We classified the samples with an Air value higher than the median Air of the full sample as the experimental group and other samples were classified as the control group. Next, we used the indicators of the firm size, ownership, leverage, interest coverage ratio, fixed assets ratio, return on total assets, growth opportunity, cash flow, provincial GDP growth rate, and proportion of provincial secondary industry to carry out a one-to-one matching of the nearest neighbors within the caliper radius (0.01), screening out the corresponding counterfactual samples, and carried out a regression. After the balanced test, the deviation of the most control variables was greatly reduced after matching, and the t-test results did not reject the original hypothesis that there was no systematic difference between the two groups. The coefficients for Air presented in Table 10 were all positive and significant at the 5% level, which proved once again that the results of the previous regression analysis were robust.

## 5. Further Analysis

### 5.1. The Moderating Role of Environmental Regulatory Pressure

Next, we explored the moderating role of environmental regulatory pressure on the relationship between air pollution and the cost of debt. As reflected in column (1) of Table 11, the coefficient on Air × PITI was positive and statistically significant at the 1% level. From this result, we concluded that environmental regulatory pressure on firms enhanced the influence of air pollution on the cost of debt. Moreover, we designated the samples into the “high PITI” partition if environmental regulatory pressure on firms was higher than the median PITI in the full sample and re-regressed Equation (1) into “high PITI” and “low PITI”. The results showed that the coefficient Air presented a significant positive correlation at the level of 1% in the “high PITI” partition in column (2), while the coefficient Air presented an insignificant correlation in the “low PITI” partition in column (3). The regression results of sub samples also showed that the impact of air pollution on the cost of debt was more significant when firms faced stronger environmental regulatory pressure.

### 5.2. The Moderating Role of Firms’ Industry Characteristics

Next, we tested the moderating role of firms’ industry characteristics. As presented in column (4) of Table 11, the coefficient of Air × Polluted was positive and statistically significant at the 5% level, which confirmed Hypothesis 3, which states that heavily polluting firms enhance the influence of air pollution on the cost of debt. Furthermore, we divided the full sample into a high polluted group and a low polluted group and re-regressed Equation (1) in both the high polluted group and the low polluted group. The coefficient on Air in column (5) presented a significant positive correlation at the level of 1% for the high polluted subsample. Conversely, the coefficient on Air in column (6) was statistically insignificant at conventional levels for the low polluted subsample. The regression results of sub samples also suggested that the influence of air pollution on the cost of debt was more pronounced for heavily polluting firms.

### 5.3. The Moderating Role of Firms’ Ownership

Our next test was the moderating role of firms’ ownership. As shown in column (1) of Table 12, the coefficient on Air × State was also positive and statistically significant at the 10% level, confirming Hypothesis 4, which states that being a state-owned firm lessens the influence of air pollution on the cost of debt. Furthermore, we divided the baseline sample into state-owned firms versus non-state-owned firms and re-regressed Equation (1) in these subsamples. The coefficient of Air in column (2) was nonsignificant in the state-owned subsample. Comparatively, the coefficient of Air in column (3) was statistically significant at the 1% level in the non-state-owned subsample. These results also confirmed that the influence of air pollution on the cost of debt was more pronounced for non-state-owned firms.

### 5.4. The Moderating Role of Firms’ Economic Contribution

Next, we tested the moderating role of firms’ economic contribution. As shown in column (4) of Table 12, the coefficient on Air × Tax was negative and statistically significant at the 5% level, confirming Hypothesis 5, which states that a firm’s economic contribution lessens the influence of air pollution on the cost of debt. Then, we divide the full sample into high tax contribution versus low tax contribution according to the median value of their tax contribution in the full sample and re-regressed Equation (1) in these subsamples. As shown in columns (5) and (6) of Table 12, the coefficient of Air was nonsignificant in the high tax contribution subsample, while the coefficient of Air was statistically significant at the 1% level in the low tax contribution subsample. These results revealed that the influence of air pollution on the cost of debt was more prominent for firms with low tax contributions.

## 6. Conclusions, Implications, Limitations, and Future Prospects

### 6.1. Conclusions

Employing a large sample of Chinese listed firms from the main board of the SSE and SZSE under different air quality conditions covering 2014 to 2018, we explored whether and how air pollution affects the cost of debt through environmental violation risk. In addition, we deeply explored the moderating role of environmental regulatory pressure, firms’ industry characteristics, firms’ ownership, and firms’ economic contribution on the relationship between air pollution and the cost of debt. Our study drew the following conclusions.

First, the air pollution of the cities where the firms were located had a positive impact on the cost of debt, and the environmental violation risk of firms was the mechanism of air pollution affecting the cost of debt. Our empirical results confirmed a positive relationship between air pollution and the cost of debt after controlling for various firm-level and provincial-level characteristics that affected the cost of debt and implementing a series of sensitivity and endogenous tests. In addition, we attempted to use the mediating effect test to confirm that environmental violation risk was the mechanism by which air pollution affected the cost of debt, which was distinct from the insights into the mechanism of credit risk and financial uncertainty [16]. Specifically, air pollution-induced environmental violation risk caused pessimistic assessments by creditors on the default risk of firms, which prompted them to raise the interest rates in terms of the loan contract to reduce the air pollution-related default loss. Overall, higher levels of air pollution led to a higher cost of debt tolerated by firms through their effects on environmental violation risk.

Second, the empirical results confirmed that environmental regulatory pressure and heavily polluting firms could strengthen the relationship between air pollution and the cost of debt. Specifically, the influence of air pollution on the cost of debt was more prominent for firms subjected to strict environmental supervision and heavily polluting firms. Strict environmental supervision and heavily polluting industry characteristics aggravated the environmental violation risk of firms caused by air pollution, eventually leading to a higher probability of debt default. Creditors chose to increase the cost of debt for firms subjected to strict environmental supervision and heavily polluting firms to mitigate the default loss associated with air pollution. This conclusion puts forward some conditions regarding increases in the adverse impact of air pollution on debt cost from the perspective of environmental supervision and corporate environmental performance that have not been tested in prior research.

Third, the empirical results confirmed that state-owned firms and firms’ economic contribution could weaken the relationship between air pollution and the cost of debt. In other words, the influence of air pollution on the cost of debt was more prominent for non-state-owned firms and firms with lower economic contributions. Since state-owned firms and regional major economic contributors have stronger bargaining power in environmental supervision, they can reduce the environmental violation risk, administrative penalty, and legal proceedings caused by air pollution to a certain extent; thus, mitigating the creditors’ concern about the debt default of these firms. This study was the first to examine the mechanism of mitigating the adverse impact of air pollution on debt cost from the perspective of ownership and firms’ economic contributions, which differs from insights on the mitigating mechanism of monitoring and the economic environment [16].

### 6.2. Implications

The results reported in our research may have critical implications for different groups. First, our research was conducive to highlight not only the importance of environmental governance for mitigating the cost of debt to firms that are exposed to air pollution, but also its importance to creditors exposed to their clients’ environmental violation risk and default risk. Specifically, firms should attach importance to environmental violation risk related to air pollution and strengthen the ability of environmental governance to reduce the cost of debt; creditors should incorporate the environmental risk signal transmitted by air pollution into their decision making to mitigate their default losses. Second, the moderating effect analysis in our study suggested that firms should judge the possible constraints of severe air pollution on debt financing based on their circumstances and heterogeneity in a timely manner. In particular, this implies that with the enhancement of environmental regulations, firms subjected to strict environmental supervision, heavily polluting firms, non-state-owned firms, and firms with low economic contributions need to be more cautious about environmental risks related to air pollution. These firms should take the initiative to strengthen their environmental governance and reduce the risk of environmental violations and pessimistic expectations of creditors for default risk through low-carbon innovation and green development, which, ultimately, reduce the cost of debt and financing constraints. Additionally, increasingly severe air pollution should suggest to government regulators that state-owned firms and regional major economic contributors may bring resistance to environmental supervision to some extent. Government regulators should strengthen supervision for environmental violations of state-owned firms and economic contributors so as to promote the long-term development of the regional economy.

### 6.3. Limitations and Future Prospects

Similar to other academic research, our empirical research also had certain limitations, which are worthy of further research. First, Zhou et al. [18] and Xu et al. [69] mentioned that media coverage is an important external mechanism affecting the response of investors or creditors to the risk of firms’ environmental violations. Media coverage intensity and media information sources regarding air pollution are likely to affect the sensitivity of creditors to the environmental violation risk of firms, which was not discussed in this research. Future research could further explore the moderating effect of media coverage on the relationship between air pollution and the cost of debt. Second, our research only evaluated the environmental violation risk of firms according to the severity of environmental violations. Creditors may attach greater importance to changes in corporate cash flow caused by environmental violations [10]. Future research could further measure the environmental violation risk based on the amounts of environmental penalties. Third, Wu et al. [1] tried to explore the effect of the six specific pollutants that constitute AQI on stock returns and trading activities. Each air pollutant may also have a different impact on the cost of debt, which was not discussed in our research. Considering that there may exist correlations between these pollutants, future research could further employ statistical methods, such as a factor analysis, principal component analysis, and cluster analysis, to merge these pollutant factors. Fourth, although our research used the OLS model that has been widely applied in many empirical analyses, parametric tests still have inherent limitations. Parameter tests need to estimate the population parameters using the population distribution and sample information, which is only applicable when the function that describes the relationship between the dependent and independent variables is known [70]. Some scholars have found that the application of parametric tests may cause model misspecification [71], which cannot correctly reveal the substantive relationship between the variables. In contrast, nonparametric tests can be applied to assess the effects of independent variables on the dependent variables in a nonlinear fashion without imposing a specific functional form, which allows the data themselves to reveal the functional relationship between the variables [70]. Future studies should further consider using nonparametric approaches to test the impact of air pollution on debt cost.

## Figures and Tables

**Table 1:** Assessment system of firms’ environmental violation risk.

Items	Category of Punishment for Environmental Violations		Score Value
1	Warning		1
2	Order to make corrections or make corrections within a time limit		1
3	Penalty	The penalty is less than 10,000 yuan	1
		The penalty is more than 10,000 yuan and less than 50,000 yuan	2
		The penalty is more than 50,000 yuan and less than 100,000 yuan	3
		The penalty is more than 100,000 yuan and less than 200,000 yuan	4
		The penalty is more than 200,000 yuan	6
4	Order to stop construction	Construction projects of registration form	3
		Construction projects of report form	6
		Construction projects of report	12
5	Order to restrict production		6
6	Order to stop production for rectification		12
7	Seal up and detain		6
8	Confiscation of illegal income and illegal property		6
9	Temporary seizure of permits or other documents		6
10	Revocation of licenses or other certificates		12
11	Environmental violation cases of administrative detention		12
12	Cases suspected of environmental crimes		12

**Table 2:** Definitions of variables.

Variables	Definition
Dependent variable	
Cost	Cost of debt, measured as the ratio of total interest expense to average interest-bearing debt
Independent variable	
Air	Air pollution, measured as the mean value of the daily AQI of the cities where the firms were located
Mediating variable	
Violation	Environmental violation risk of firms, measured as Ln (Severity + 1)
Control variables	
Size	Natural logarithm of total assets at the end of year
State	0 if the affiliation of the actual controller of a firm is the state, and 1 otherwise
Lev	Ratio of total liabilities to total assets at the end of the year
Ic	Interest coverage ratio, measured as the ratio of EBIT to interest expense
Fix	Ratio of total fixed assets to total assets at the end of year
Roa	Return on assets, measured as the ratio of EBIT to average total assets
Growth	Growth rate of a firm’s total revenue from year t − 1 to year t
Cfo	Ratio of net cash flow from operating activities to total assets at the end of year
Gdp	Growth rate of a province’s total GDP from year t − 1 to year t
Second	Proportion of secondary industry in the province where the firms were located

Notes: AQI was the air quality index; Severity was the severity of environmental violations; EBIT was the earnings before interest and tax; GDP was the gross domestic product.

**Table 3:** Air pollution conditions of the provinces where the sample firms were located.

Province	Mean AQI	N	Province	Mean AQI	N
Hebei	124.1973	99	Hunan	85.2114	111
Henan	115.0412	146	Jilin	84.2591	77
Beijing	111.4179	354	Inner Mongolia	83.9621	59
Shaanxi	109.8092	69	Heilongjiang	82.3223	70
Tianjin	107.3100	60	Chongqing	82.2441	80
Xinjiang	105.8833	84	Shanghai	82.0048	307
Shandong	104.0479	267	Zhejiang	78.8685	351
Shanxi	101.2360	108	Jiangxi	71.6547	72
Gansu	97.1313	49	Guangxi	66.4132	60
Hubei	96.4351	150	Guizhou	63.4468	44
Ningxia	96.0047	29	Tibet	63.0077	16
Jiangsu	90.9829	304	Guangdong	62.9685	343
Sichuan	90.9101	176	Fujian	57.5179	101
Qinghai	86.8484	32	Yunnan	56.5209	61
Anhui	86.8321	146	Hainan	46.4630	34
Liaoning	86.2856	134	Total	88.7361	3993

Notes: Mean AQI was the mean values of the daily air quality index during the sample period.

**Table 4:** Variable descriptive statistics.

Variables	Obs.	Mean	Std. Dev.	Min	Median	Max
Cost	3993	0.0320	0.0157	0.0004	0.0314	0.0773
Air	3993	88.7361	20.9627	46.4630	86.7135	142.000
Size	3993	22.9095	1.3719	20.1914	22.7688	26.8076
State	3993	0.3869	0.4871	0	0	1
Lev	3993	0.5173	0.1824	0.1207	0.5179	0.9450
Ic	3993	16.2714	49.4600	−16.7000	4.0300	388.0000
Fix	3993	0.2967	0.1916	0.0103	0.2673	0.7888
Roa	3993	0.0520	0.0562	−0.1487	0.0468	0.2313
Growth	3993	0.1625	0.4536	−0.4920	0.0849	3.0500
Cfo	3993	0.0474	0.0626	−0.1325	0.0459	0.2331
Gdp	3993	0.0769	0.0432	−0.2240	0.0823	0.1459
Second	3993	0.4104	0.0874	0.1863	0.4399	0.5313

Notes: Cost was the cost of debt; Air was the air pollution; Size was the size of firms; State was the ownership of firms; Lev was the leverage of firms; Ic was the interest coverage ratio of firms; Fix was the fixed assets ratio of firms; Roa was the return on total assets; Growth was the revenue growth rate of firms; Cfo was the operating cash flow of firms; Gdp was the provincial GDP growth rate; Second was the proportion of provincial secondary industry.

**Table 5:** Pearson correlations.

Variables	Cost	Air	Size	State	Lev	Ic	Fix	Roa	Growth	Cfo	Gdp	Second
Cost	1.0000											
Air	0.0084	1.0000										
Size	0.0734 ***	0.1175 ***	1.0000									
State	−0.0101	0.1862 ***	0.2975 ***	1.0000								
Lev	0.1345 ***	0.1202 ***	0.3795 ***	0.2388 ***	1.0000							
Ic	0.3158 ***	−0.0232	0.0753 ***	0.0651 ***	0.2649 ***	1.0000						
Fix	0.2994 ***	0.0391 **	0.1378 ***	0.2073 ***	0.0701 ***	0.1231 ***	1.0000					
Roa	−0.0401 **	0.0476 ***	0.0584 ***	0.1450 ***	0.3310 ***	0.3035 ***	−0.0321 **	1.0000				
Growth	0.0214	−0.0240	0.0036	0.0954 ***	0.0462 ***	0.0508 ***	0.0870 ***	0.2420 ***	1.0000			
Cfo	0.0793 ***	−0.0224	0.1299 ***	−0.0197	0.1379 ***	0.1313 ***	0.3237 ***	0.4049 ***	−0.0109	1.0000		
Gdp	0.0827 ***	0.0571 ***	−0.0135	0.0577 ***	0.0624 ***	0.0045	0.0800 ***	0.0610 ***	0.0308 *	0.0162	1.0000	
Second	0.0784 ***	0.0871 ***	0.2299 ***	0.0801 ***	−0.0208	−0.0312 **	0.1013 ***	0.0188	−0.0089	0.0128	−0.0265 *	1.0000

Notes: The explanation for abbreviations of all variables used inside Table 5 were mentioned in the footer of Table 4. *, **, and *** reflect *p*-values of the correlation coefficients between variables were less than 0.1, 0.05, and 0.01, respectively.

**Table 6:** Influence of air pollution on cost of debt and mechanism of environmental violation risk.

Variables	(1) Cost	(2) Cost	(3) Violation	(4) Cost
Air	0.0000 ** (2.42)	0.0001 *** (3.02)	0.0044 *** (3.38)	0.0001 *** (2.73)
Violation				0.0006 ** (2.06)
Size		0.0003 (1.60)	0.0834 *** (6.01)	0.0002 (0.69)
State		0.0036 *** (7.06)	−0.0867 ** (−2.43)	0.0044 *** (7.91)
Lev		0.0085 *** (5.90)	0.0425 (0.44)	0.0084 *** (5.50)
Ic		−0.0001 *** (−17.28)	−0.0001 (−0.19)	−0.0001 *** (−14.71)
Fix		0.0119 *** (7.63)	0.4438 *** (4.10)	0.0104 *** (6.11)
Roa		0.0078 (1.64)	−0.1507 (−0.46)	0.0093 * (1.82)
Growth		0.0025 *** (5.32)	−0.0087 (−0.27)	0.0027 *** (5.41)
Cfo		0.0033 (0.83)	0.0770 (0.28)	0.0058 (1.35)
Gdp		−0.0015 (−0.23)	0.2689 (0.61)	−0.0015 (−0.21)
Second		0.0021 (0.13)	−0.1988 (−0.18)	0.0076 (0.44)
_Cons	0.0205 *** (4.00)	0.0030 (0.29)	−1.0131 (−1.43)	0.0011 (0.10)
Year	Yes	Yes	Yes	Yes
Industry	Yes	Yes	Yes	Yes
Province	Yes	Yes	Yes	Yes
F-statistics	15.57 ***	22.08 ***	9.22 ***	18.76 ***
Adjusted-R^2^	0.2346	0.3318	0.1832	0.3288
N	3993	3993	3372	3372
Sobel-test			1.7600 *	

Notes: Columns (1) and (2) represent the regression results on the influence of air pollution on the cost of debt. Columns (3) and (4) represent the regression results on the mechanism of environmental violation risk. Violation was the environmental violation risk of firms. The explanation for abbreviations of other variables used inside Table 6 were mentioned in the footer of Table 4. The numbers in brackets are *t*-values. *, **, and *** reflect *p*-values of the coefficients were less than 0.1, 0.05, and 0.01, respectively.

**Table 7:** Analysis of variance (ANOVA).

**Model (1)**					
**Source**	**SS**	**df**	**MS**	**F-Statistics**	**Sig.**
Model	0.2452	84	0.0029	15.57	0.0000
Residual	0.7329	3908	0.0002		
Total	0.9781	3992	0.0002		
**Model (2)**					
**Source**	**SS**	**df**	**MS**	**F-Statistics**	**Sig.**
Model	0.3399	94	0.0036	22.08	0.0000
Residual	0.6383	3898	0.0002		
Total	0.9781	3992	0.0002		

**Table 8:** Sensitivity test for measurement of air pollution and the cost of debt.

Variables	(1) Cost	(2) Cost	(3) Expense	(4) Expense	(5) Expense
Air			0.0000 *** (2.83)		
PM_2.5	0.0001 *** (3.57)			0.0001 *** (3.25)	
AQI_grade		0.0016 ** (2.39)			0.0015 *** (2.72)
Size	0.0003 * (1.79)	0.0003 (1.61)	0.0003 ** (2.15)	0.0004 ** (2.19)	0.0003 ** (2.16)
State	0.0039 *** (7.44)	0.0036 *** (6.94)	0.0034 *** (8.12)	0.0037 *** (8.53)	0.0034 *** (8.03)
Lev	0.0074 *** (5.00)	0.0085 *** (5.90)	0.0101 *** (8.59)	0.0096 *** (7.84)	0.0101 *** (8.58)
Ic	−0.0001 *** (−17.05)	−0.0001 *** (−17.34)	−0.0001 *** (−18.09)	−0.0001 *** (−17.84)	−0.0001 *** (−18.16)
Fix	0.0115 *** (7.28)	0.0119 *** (7.59)	0.0136 *** (10.62)	0.0130 *** (10.01)	0.0136 *** (10.59)
Roa	0.0068 (1.40)	0.0079 * (1.67)	0.0051 (1.30)	0.0042 (1.05)	0.0052 (1.33)
Growth	0.0026 *** (5.28)	0.0026 *** (5.36)	0.0017 *** (4.38)	0.0017 *** (4.26)	0.0017 *** (4.42)
Cfo	0.0032 (0.78)	0.0033 (0.83)	0.0008 (0.26)	0.0012 (0.35)	0.0009 (0.26)
Gdp	−0.0020 (−0.31)	−0.0009 (−0.14)	−0.0018 (−0.33)	−0.0019 (−0.35)	−0.0015 (−0.28)
Second	0.0066 (0.40)	−0.0001 (−0.01)	−0.0003 (−0.03)	0.0013 (0.10)	−0.0024 (−0.18)
_Cons	−0.0008 (−0.07)	0.0061 (0.58)	−0.0021 (−0.24)	−0.0034 (−0.39)	−0.0002 (−0.02)
Year	Yes	Yes	Yes	Yes	Yes
Industry	Yes	Yes	Yes	Yes	Yes
Province	Yes	Yes	Yes	Yes	Yes
F-statistics	21.54 ***	22.03 ***	25.80 ***	24.88 ***	25.79 ***
Adjusted-R^2^	0.3357	0.3312	0.3687	0.3701	0.3686
N	3822	3993	3993	3822	3993

Notes: Columns (1) and (2) represent the regression results on the sensitivity test of air pollution. Columns (3), (4), and (5) represent the regression results on the sensitivity test of the cost of debt. Cost was the ratio of total interest expense to average interest-bearing debt; Expense was the ratio of total interest expense to average total debt; Air was the average air quality index; PM_2.5 was the average PM_2.5_ concentration; AQI_grade was the grade of average air quality index. The explanation for abbreviations of other variables used inside Table 8 were mentioned in the footer of Table 4. The numbers in brackets are *t*-values. *, **, and *** reflect *p*-values of the coefficients were less than 0.1, 0.05, and 0.01, respectively.

**Table 9:** Endogenous test of 2SLS analysis.

Variables	(1) Step 1 Air	(2) Step 2 Cost
Air		0.0003 *** (2.75)
Rain	−0.0018 ** (−2.26)	
Wind	−5.2605 *** (−8.38)	
Size	−0.0174 (−0.11)	0.0003 (1.53)
State	−1.9900 *** (−4.79)	0.0042 *** (7.21)
Lev	0.2585 (0.21)	0.0084 *** (5.10)
Ic	0.0001 (0.03)	−0.0001 *** (−14.65)
Fix	−0.6003 (−0.48)	0.0122 *** (6.79)
Roa	0.9495 (0.22)	0.0077 (1.33)
Growth	0.0934 (0.24)	0.0025 *** (3.58)
Cfo	0.1855 (0.06)	0.0033 (0.72)
Gdp	31.2401 *** (5.36)	−0.0095 (−1.28)
Second	−9.8322 (−0.58)	0.0026 (0.15)
_Cons	113.4483 *** (11.68)	−0.0215 (−1.36)
Year	Yes	Yes
Industry	Yes	Yes
Province	Yes	Yes
F-statistics	200.27 ***	
Wald		10187.56 ***
R^2^	0.7562	0.3176
N	3993	3993

Notes: Table 9 reflects the results on the endogenous test of 2SLS analysis. Rain was the rainfall of the cities; Wind was the wind speed of the cities. The explanation for abbreviations of other variables used inside Table 9 were mentioned in the footer of Table 4. In particular, Rain and Wind were tool variables used in 2SLS analysis. ** and *** reflect *p*-values of the coefficients were less than 0.05 and 0.01, respectively.

**Table 10:** Endogenous test of PSM estimation.

Variables	(1) Cost	(2) Cost
Air	0.0001 ** (2.30)	0.0001 ** (2.53)
Size		0.0001 (0.50)
State		0.0043 *** (6.12)
Lev		0.0100 *** (4.98)
Ic		−0.0001 *** (−11.17)
Fix		0.0102 *** (4.62)
Roa		0.0038 (0.57)
Growth		0.0025 *** (3.93)
Cfo		0.0110 ** (2.01)
Gdp		0.0022 (0.24)
Second		0.0069 (0.30)
_Cons	0.0254 *** (3.30)	0.0078 (0.52)
Year	Yes	Yes
Industry	Yes	Yes
Province	Yes	Yes
F-statistics	9.72 ***	12.89 ***
Adjusted-R^2^	0.2568	0.3456
N	2095	2095

Notes: Table 10 reflects the results on the endogenous test of PSM estimation. The explanation for abbreviations of all variables used inside Table 10 were mentioned in the footer of Table 4. ** and *** reflect *p*-values of the coefficients were less than 0.05 and 0.01, respectively.

**Table 11:** Moderating effect of firm’s environmental regulatory pressure and industry characteristic.

Variables	(1) Cost	(2) Cost	(3) Cost	(4) Cost	(5) Cost	(6) Cost
	Full Sample	High PITI	Low PITI	Full Sample	High Polluted	Low Polluted
Air	0.0001 *** (3.43)	0.0001 *** (3.26)	0.0000 (0.89)	0.0001 *** (2.76)	0.0001 *** (3.31)	0.0000 (1.32)
Air * PITI	0.0003 *** (2.93)					
Air * Polluted				0.0000 ** (2.25)		
PITI	−0.0029 (−0.96)					
Polluted				0.0184 *** (4.04)		
State	0.0037 *** (7.14)	0.0021 *** (2.91)	0.0051 *** (6.62)	0.0036 *** (7.05)	0.0036 *** (4.92)	0.0036 *** (4.95)
Size	0.0003 (1.51)	0.0003 (1.28)	0.0002 (0.66)	0.0003 (1.51)	0.0004 (1.27)	0.0001 (0.52)
Lev	0.0084 *** (5.85)	0.0100 *** (4.81)	0.0074 *** (3.62)	0.0086 *** (5.95)	0.0109 *** (5.59)	0.0068 *** (3.18)
Ic	−0.0001 *** (−17.31)	−0.0001 *** (−12.05)	−0.0001 *** (−11.91)	−0.0001 *** (−17.19)	−0.0001 *** (−12.28)	−0.0001 *** (−11.68)
Fix	0.0119 *** (7.59)	0.0093 *** (4.14)	0.0150 *** (6.68)	0.0117 *** (7.44)	0.0132 *** (6.19)	0.0088 *** (3.66)
Roa	0.0078 (1.64)	0.0069 (1.03)	0.0120 * (1.77)	0.0077 (1.62)	0.0096 (1.53)	0.0016 (0.22)
Growth	0.0025 *** (5.34)	0.0039 *** (5.92)	0.0014 ** (2.04)	0.0026 *** (5.38)	0.0041 *** (5.89)	0.0011 (1.63)
Cfo	0.0030 (0.76)	−0.0045 (−0.81)	0.0094 * (1.67)	0.0035 (0.88)	0.0108 * (1.95)	−0.0016 (−0.28)
Gdp	−0.0004 (−0.07)	−0.0072 (−0.58)	−0.0037 (−0.41)	−0.0013 (−0.20)	−0.0035 (−0.43)	−0.0007 (−0.07)
Second	0.0077 (0.46)	0.0962 (1.94)	−0.0030 (−0.15)	0.0012 (0.08)	0.0182 (0.86)	−0.0151 (−0.58)
_Cons	0.0057 (0.54)	−0.0273 (−1.02)	0.0105 (0.79)	0.0179 * (1.84)	0.0049 (0.37)	0.0240 (1.57)
Year	Yes	Yes	Yes	Yes	Yes	Yes
Industry	Yes	Yes	Yes	Yes	Yes	Yes
Province	Yes	Yes	Yes	Yes	Yes	Yes
F-statistics	21.78 ***	13.74 ***	11.97 ***	21.93 ***	14.01 ***	14.57 ***
Adjusted-R^2^	0.3332	0.3330	0.3329	0.3324	0.2983	0.3317
N	3993	1991	2002	3993	2051	1942

Notes: Columns (1) to (3) of this table represent the results on the moderating role of environmental regulatory pressure. Columns (4) to (6) of this table represent the results on the moderating role of industry characteristic. PITI was the Pollution Information Transparency Index; Polluted was the firms’ industry characteristics. The explanation for abbreviations of other variables used inside Table 11 were mentioned in the footer of Table 4. The numbers in brackets are *t*-values. *, **, and *** reflect *p*-values of the coefficients were less than 0.1, 0.05, and 0.01, respectively.

**Table 12:** Moderating effect of firm’s ownership and economic contribution.

Variables	(1) Cost	(2) Cost	(3) Cost	(4) Cost	(5) Cost	(6) Cost
	Full Sample	State-Owned	Non-State-Owned	Full Sample	High Tax Contribution	Low Tax Contribution
Air	0.0001 *** (3.06)	0.0000 (1.61)	0.0001 *** (2.79)	0.0001 *** (2.91)	−0.0000 (−0.38)	0.0001 *** (2.60)
Air * State	0.0000 * (1.75)					
Air * Tax				−0.0031 ** (−2.48)		
Tax				−0.0887 *** (−2.35)		
State	0.0037 *** (7.16)			0.0037 *** (7.20)	0.0030 *** (4.14)	0.0046 *** (6.17)
Size	0.0003 (1.58)	0.000 * (1.91)	0.0001 (0.14)	0.0006 *** (2.90)	−0.0004 (−1.30)	0.0015 *** (3.64)
Lev	0.0085 *** (5.93)	0.0079 *** (4.59)	0.0118 *** (4.52)	0.0080 *** (5.55)	0.0133 *** (6.44)	0.0084 *** (4.11)
Ic	−0.0001 *** (−17.30)	−0.0001 *** (−12.08)	−0.0001 *** (−10.18)	−0.0001 *** (−17.21)	−0.0001 *** (−11.86)	−0.0001 *** (−11.92)
Fix	0.0119 *** (7.62)	0.0086 *** (4.67)	0.0208 *** (6.75)	0.0121 *** (7.78)	0.0139 *** (6.49)	0.0126 *** (5.31)
Roa	0.0077 (1.63)	0.0146 ** (2.44)	0.0001 (0.01)	0.0080 * (1.69)	0.0173 ** (2.47)	0.0068 (1.03)
Growth	0.002 *** (5.25)	0.0026 *** (4.47)	0.0023 *** (3.00)	0.0025 *** (5.18)	0.0050 *** (7.99)	0.0004 (0.50)
Cfo	0.0034 (0.86)	0.0133 *** (2.77)	−0.0084 (−1.26)	0.0044 (1.10)	0.0070 (1.25)	0.0003 (0.05)
Gdp	−0.0013 (−0.20)	0.0035 (0.50)	−0.0073 (−0.54)	−0.0012 (−0.18)	−0.0021 (−0.29)	−0.0004 (−0.03)
Second	0.0021 (0.13)	0.0032 (0.17)	−0.0068 (−0.24)	0.0003 (0.02)	0.0238 (1.26)	−0.0231 (−0.83)
_Cons	0.0100 (0.97)	0.0192 (1.51)	−0.0108 (−0.57)	0.0021 (0.19)	−0.0138 (−1.00)	0.0029 (0.16)
Year	Yes	Yes	Yes	Yes	Yes	Yes
Industry	Yes	Yes	Yes	Yes	Yes	Yes
Province	Yes	Yes	Yes	Yes	Yes	Yes
F-statistics	21.89 ***	18.63 ***	9.29 ***	21.86 ***	17.56 ***	10.38 ***
Adjusted-R^2^	0.3321	0.3881	0.3235	0.3341	0.4276	0.3018
N	3993	2448	1545	3993	1996	1997

Notes: Columns (1) to (3) represent the regression results on the moderating role of ownership. Columns (4) to (6) represent the regression results on the moderating role of economic contribution. Tax was the economic contribution of firms. The explanation for abbreviations of other variables used inside Table 12 were mentioned in the footer of Table 4. The numbers in brackets are *t*-values. *, **, and *** reflect *p*-values of the coefficients were less than 0.1, 0.05, and 0.01, respectively.

## Data Availability

The data shown in this research are available on request from the corresponding author.
